# Efficacy of physical activity shared between parents and children to improve sports initiation in the M.A.M.I.deporte® program

**DOI:** 10.3389/fspor.2024.1372664

**Published:** 2024-03-26

**Authors:** Belén Cueto-Martín, Juan Carlos De la Cruz-Márquez, Rafael Burgueño-Menjíbar, Eduardo García-Mármol

**Affiliations:** ^1^ Department of Physical Education and Sports, Faculty of Sport Sciences, University of Granada, Granada, Spain; ^2^ Department of Didactics of Languages, Arts and Sports, University of Málaga, Málaga, Spain; ^3^ Department of Physical Education and Sports, Faculty of Education and Sport Sciences, University of Granada, Melilla, Spain

**Keywords:** sport, childhood, initiation, intervention, parents, joint participation, Sport drop out

## Abstract

**Objective:**

To determine if the active methodology for improving sports initiation (M.A.M.I.deporte®) shared between children and parents successfully promotes children in sports activities, maintains their activity and improves long-term adherence.

**Participants:**

The study involved 118 participants aged between 2 and 11 years (6.3 ± 2.3). In the first season, 34 participated (16 girls; 18 boys); in the second season, 46 participated (22 girls; 24 boys) and in the third season, 38 participated (19 girls; 19 boys).

**Methodology:**

It was carried out from October to June over three academic years for two hours a week. Every 4 sessions a different sporting activity was carried out, planned so that parents and children could practise them, simultaneously.

**Analysis:**

At the beginning and end of each period, a survey was carried out on the sports activities in which the participants had started. If participants remained in the activity, the survey was face-to-face and if participants no longer attended the activity, they were contacted by telephone. Descriptive values were obtained for the variables in absolute and percentage form and a repeated measures anova was performed.

**Results:**

Vigorous physical activity performed was 3.82 ± 1.16 h/week in the first year, 3.38 ± 1.59 in the second year and 2.99 ± 1.46 in the third year with no significant differences between any of the years. 32.20% joined other sporting activities and only 6.78% gave up vigorous physical activity.

**Conclusion:**

Joint activity of parents and children contributed to maintaining vigorous physical activity at the recommended levels in the child population with only 6.78% (*n *= 8) of the participants dropping out.

## Introduction

Promoting physical activity (PA) during childhood and adolescence is considered an appropriate strategy for fostering the acquisition and consolidation of healthy habits throughout a person's life (1). In this regard, parents could play a central role in the PA of children and adolescents (2). For this reason, it is believed that the family could be a relevant target population for interventions aimed at increasing PA levels in the child population.

More specifically, research has consistently documented that parents' support behaviors toward PA have been positively associated not only with leisure-time PA in children and adolescents (3, 4, 5) but also with motivational precursors of PA behavior, such as self-determined motivation and motor self-efficacy exhibited by children and adolescents (6, 7), as well as their autonomy, relationship, and attitudes toward leisure-time PA (7). In the same vein, previous studies have shown that paternal support was more strongly related to PA in adolescents than maternal support, although both supports were equally associated with self-determined motivation and intention toward PA (8). In the case of the child population, previous works have indicated that maternal support was more strongly related to PA than paternal support (9). In addition to parental support, previous research has also shown that when both parents are physically active, the likelihood of the persistence of PA over time doubles for male children and adolescents, while it multiplies between three and eight times for female children (10, 11).

To date, the majority of family-focused interventions have centered on the development of physical exercise programmes, both at home (12, 13, 14) and in outdoor spaces (15), to promote PA among all members of the family unit. In these studies, there was an initial training phase for parents on basic PA guidelines, and they were responsible for leading the programme for the entire family in a later phase. On the other hand, a limited number of interventions have focused on developing exercise programmes exclusively for specific family members, such as maternal grandmothers, mothers and daughters (16), or fathers and daughters (17). However, there is limited evidence regarding family intervention programmes that aim to promote PA through sports activities practiced jointly and simultaneously by parents and children.

In previous studies, we have demonstrated significant differences in the levels of intrinsic motivation, integrated regulation, identified regulation, and introjected regulation of parents in favor of shared PA between parents and children, without gender influence (18). Other works have reported an increase in the level of physical fitness for all family members (19, 20), better body composition, as well as increased satisfaction with sports participation (21).

The rationale for our project is based on studies that have reported a gradual decline in children's physical exercise practice in relation to age, confirming high dropout rates in the adolescent period (22, 23, 24). Such evidence of low levels of physical activity, declining participation in physical activity from childhood to adolescence, reduced participation in regular sport among young people and numerous adverse outcomes associated with participation (25) suggest the need to examine the reasons for these discouraging trends but also to implement physical activity programmes that reinforce children's participation and are sustained over time (26).

The aim was to determine whether the M.A.M.I. Deporte programme of shared PA between children and parents over three school years successfully promotes children's sport practice, maintains their activity and improves long-term adherence. Parents are considered to play a key role in children's adherence to physical activity and it is important to promote PA in the family for the improvement of children's PA (27, 28, 29) as demonstrated by some family-based physical activity promotion programmes (30) although there is little evidence of the effectiveness of family engagement methods in children's PA promotion programmes, due to heterogeneity in study design, study quality and outcome measures used (31) and so there appears to be a need to study models of joint family intervention that are more predictive of children's physical activity. Against this background, the hypothesis of our work is that joint parent-child participation in the MAMI Deporte programme maintains PA in the long term and encourages children to participate in other vigorous sporting activities.

## Material and methods

### Design

A longitudinal non-experimental design of multiple observations was carried out in which a single group of subjects consisting of parents and children was measured for the same dependent variable, physical activity practice, at the same time intervals (one school year), in order to determine the permanence in the MAMI Sport programme. In this research, none of the participants were pre-selected; they became aware of the M.A.M.I. Deporte family sports activity programme through local written communications, posters, web resources, and interpersonal oral communication, and chose to participate voluntarily. Activities were carried out simultaneously by parents, mothers, and children.

### Participants

A total of 64 families participated in this study. The sample was selected using a non-probability technique of snowball sampling due to the difficulty encountered in previous exploratory studies in finding a representative sample of participants (Tables 1, 2).

**Table 1:** Number of participants per year and their characterisation.

	Girls	Boys
First year	16	18
Second year	22	24
Third year	19	19
Three-year total	57	61
	M ± SD	M ± SD
Age	6.90 ± 2.20	5.60 ± 2.20
Height (cm)	120 ± 0.16	1.15 ± 0.16
Weight (Kg)	26.00 ± 9.10	23.00 ± 10.10
BMI (Kg/m2)	17.80 ± 0.93	17.24 ± 1.87

M ± SD: mean and standard deviation.

**Table 2:** Sample distribution by age.

Age	Girls	Boys		
	*N*	%	*n*	%
2	2	1.69	2	1.69
3	5	4.24	4	3.39
4	5	4.24	5	4.24
5	9	7.63	15	12.71
6	11	9.32	16	13.56
7	8	6.78	3	2.54
8	7	5.93	5	4.24
9	5	4.24	3	2.54
10	4	3.39	4	3.39
11	1	0.85	4	3.39
TOTAL	57	48.31	61	51.69

%: Percentage.

Identifying data (age, weight, and height) were collected in the month of October for each of the three years.

### Procedure

Informed consent was obtained from the parents of underage participants to allow them to complete the survey, as well as to permit electronic or telephone contact, even if they were no longer participating in the program. All personal data were safeguarded by the research team, using them exclusively for scientific purposes in accordance with the Spanish Personal Data Protection Law (Organic Law 15/1999, of December 13). The study was approved by the University of Granada Ethics Committee (approval code: 322/CEIH/2017).

In October and June of each school year, each participant completed a survey related to sports activities they had initiated during the M.A.M.I. Deporte programme. If participants continued attending the programme, the survey was conducted in person. If these participants no longer attended the programme, they were contacted by phone to provide retrospective answers. For this group of participants who no longer attended the program, information was requested about the type of sports activity they engaged in, weekly practice hours, activity level, participation in competitions, and the duration of their engagement in that sports activity. This telephone interview was conducted by the principal investigator.

### Intervention programme

The detailed methodology has been described in previous studies (18, 20, 21). The M.A.M.I. Deporte family sports activity programme took place from October to June over three academic years: Year 1 (29 sessions, 58 h), Year 2 (29 sessions, 58 h), and Year 3 (32 sessions, 64 h), totaling 90 sessions and 180 h of practice. The sessions were scheduled for 2 h every Friday to facilitate participation without hindering other extracurricular activities.

#### Planning

Each of the following sports was addressed during four consecutive sessions: handball, basketball, soccer, combat sports, rhythmic and artistic gymnastics, swimming, volleyball, and athletics. The sports planning strategy was based on specific guidelines: a) Activities began with three team sports followed by three individual sports. Toward the end of the programme, both types were reinforced with the practice of one team sport and one individual sport; b) It started with manual-dominant team sports to encourage group integration, interaction with peers (parents/mothers and children from different families), and segmental coordination based on eye-hand regulation. The programme continued with the practice of a team sport with foot dominance to increase the complexity of motor actions and involve more participants in collective and technical-tactical actions; c) Individual sports were then introduced, combining interaction skills with opponents, mobile equipment, and aquatic environments; d) The annual plan concluded with a team sport to strengthen group cohesion and an individual sport that allowed for individual achievements.

#### Implementation

To carry out the family sports activity programme, a mixed strategy was adopted, including games aimed at improving technical and tactical components, as well as real-game situations and competition.

Regarding parents, the goal was to fully integrate them into the practice, not only with their own children but also to interact with all participating children to promote collaboration among parents with all team members. In addition to playing a role in practice, parents were trained in a specific activity, which they then taught to the children immediately afterward. It was a priority to ensure that the activity level of parents was not diminished, balancing the number of children and adults in the activities.

In each session, the objectives, work methodology, and activities were explained at the beginning. Each session was divided into three distinct parts. The first part involved knowledge games where families shared their names, sports preferences, dietary habits, and recreational interests to strengthen mutual understanding and affection. Participants could take on the roles of players, collaborators, or spectators, choosing one role at a time. In the second part of the session, different skills of the sport scheduled for that month were practiced. The first session of each month approached the sport from a playful perspective, while the next three sessions delved into both technical-tactical fundamentals and regulations. In this regard, each child was required to bring a known or noted rule to share with the rest of the group. The third part of the session aimed to promote healthy eating habits. For this purpose, parents brought healthy snacks, such as water, natural juices without added sugars, sugar-free whole milk shakes, seasonal fruits, and homemade sandwiches, avoiding all types of pastries, processed meats, sugars, chocolates, or similar items.

### Statistical analysis

The statistical analysis was carried out using IBM SPSS Statistics for Windows (version 28.0, IBM Corp, Armonk, NY, USA). Descriptive values of variables were obtained in both absolute and percentage terms. Both a normality analysis (Shapiro-Wilk test) and a Mahchly test were carried out, obtaining that the distribution of hours of PA per year conformed to a normal distribution, with a *p* > .05, and that the hours of PA met the standard of sphericity (*p* > .05). An analysis of repeated measures of variance (ANOVAs) was used to analyze the PA hours in the three years. Statistical significant effects were further analysis by paired-sample t-test corrected by Holm-Bonferroni comparison. Effects size is indicated with Coheńs d for *t*-test and partial eta squared for Fs.

## Results

Table 3 shows that in the first year of the M.A.M.I. Deporte programme, a total of 34 children participated, of whom 12 (35.29%) only engaged in PA within this sports activity programme, while another 23 (64.71%) children also participated in other sports activities during the week. Of these 34 children, only 23 (67.64%) participated in the second year of this programme, among whom 11 (32.35%) solely practiced PA in M.A.M.I. Deporte, while 8 (23.53%) had started other organized sports activities, and 3 (8.82%) did not engage in any other PA. In the third year of the programme, out of the initial 34 children, 14 (41.17%) continued, while 18 (52.94%) engaged in other sports activities, and 2 (5.88%) completely discontinued PA.

**Table 3:** Evolution of participation.

YEAR	FIRST	SECOND			THIRD			GLOBAL		
	Participants since the first year									
	*n*_1_	*n*_2_	Other PA	Dropout	*n*_3_	Other PA	Dropout	*n*_g_	Other PA	Dropout
Participating by year	34	23	8	3	14	18	2			
	Participants since the second year									
		23			8	12	3			
	Participants since the third year									
					16					
	Total per season									
	34	46	8	3	38	30	5	118	38	8
%	28.81	38.98	6.78	2.54	32.20	25.42	4.24	100.00	32.20	6.78

*n_n_* = number of participants per annual season.

Other PA = participants who did not participate in the MAMI Deporte programme but engaged in another PA.

No PA = participants who abandoned both the MAMI Deporte programme and any other PA.

In the second year, 23 children joined the M.A.M.I. Deporte programme, of whom 8 (34.78%) continued in the following year. Additionally, 12 (52.17%) children started other sports activities, and 3 (13.04%) children did not engage in any form of PA. In the third year of the M.A.M.I. Deporte programme, 16 children joined, of which 10 (62.50%) were involved in another PA, and 6 (37.50%) only attended this programme.

Table 4 shows that boys and girls who started the M.A.M.I. Deporte programme in the first year engaged in vigorous PA for 3.82 h per week (h/week), which decreased to 3.59 ± 1.82 h in the third year. Overall, vigorous PA averaged 3.20 ± 1.40 h/week over the three years analyzed in the M.A.M.I. Deporte programme.

**Table 4:** Hours of vigorous physical activity per week.

YEAR	FIRST	SECOND	THIRD	TOTAL
Boys and girls who attended since the first year				
*M* ± S*D*	3.82 ± 1.60	3.62 ± 1.88	3.32 ± 1.98	3.59 ± 1.82
Boys and girls who attended since the second year				
*M* ± S*D*		3.13 ± 1.29	2.39 ± 1.34	2.76 ± 1.32
Boys and girls who attended since the third year				
*M* ± S*D*		3.25 ± 1.06	3.25 ± 1.06	
Total				
Total Hours	3.82 ± 1.60	3.38 ± 1.59	2.99 ± 1.46	3.20 ± 1.40

M ± SD: mean and standard deviation.

At the beginning of the second and third year, one of the research team members telephoned the parents of the children who dropped out of the programme (6.78%; *n *= 8) (Table 5) to inquire about the circumstances of the drop out. In most cases, the most frequently cited reason was the parents' inability to accompany their children because of work or because they had to take care of other family members.

**Table 5:** Drop out rates by age and gender.

Age	Girls		Boys	
	*n*	%	*n*	%
8	2	1.69		
9	2	1.69	1	0.85
10	1	0.85	2	1.69
Total	5	4.23	3	2.55

%: Percentage.

**Figure 1:**
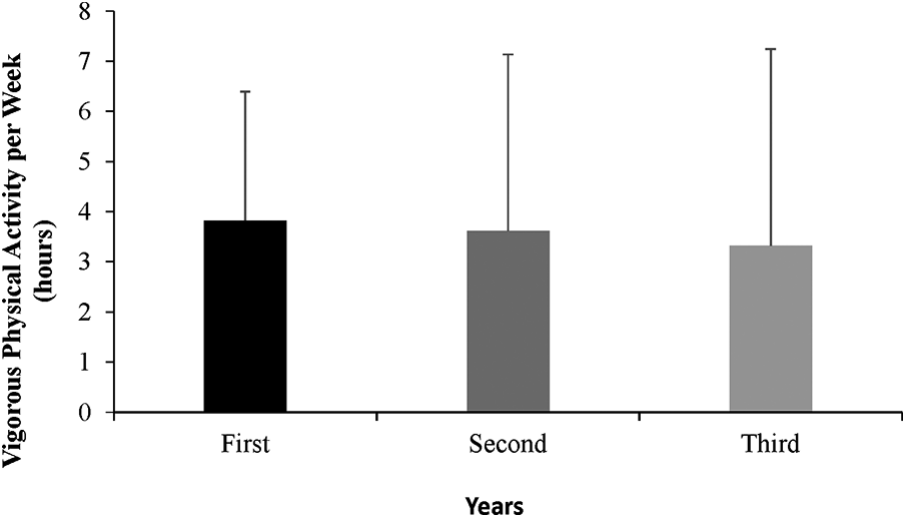
PA per weeks (hours, average ± SD).

A repeated measures ANOVA were employed for obtaining differences between PA hours in the three years. Paired t-tests also showed that there was no difference between the means of weekly vigorous physical activity between years. In this sense, dataset revealed no significant differences, *F *= 1.20, *p* = .31, *η*2 = .04. In all case Figure 1 shows the hours (averages and SD) PA per week. The average of the two groups is decreasing, with no significant difference between them. Finally, no post-hoc analysis was necessary.

## Discussion

The purpose of the M.A.M.I. Deporte programme was to promote children's engagement in PA through a joint participation program with their parents. Over the three academic years in which this PA program was implemented, it is noteworthy that 32.20% of children joined other sports activities, but of a federative or competitive nature, while only 6.78% discontinued both the sports activity program and leisure-time PA. All of them were over 8 years old and well beyond the fundamental movement stage of Gallahue and should already be adapted to school (32). The majority of children continued in the M.A.M.I. Deporte programme, alongside participating in other sports activities.

Conceptually, the MAMI Deporte Methodology can be included within the theory of physical literacy, promoting Motivation, confidence, physical competence, knowledge and understanding of the value and taking responsibility for adhering to physical activity in life (33).

In the first year, participants engaged in vigorous PA for 3.82 ± 1.60 h per week (h/week), which no significantly decreased to 3.32 ± 1.98 in the third year (Table 4). This reduction (0.50 h/week) represents only a 30-minute decrease in weekly sports activities. However, children who joined in the second year no significantly decreased their vigorous PA in the third year (3.13 ± 1.29 vs. 2.39 ± 1.34 h/week), indicating a decrease of 0.74 h/week but it was also not statistically significant.

These results may appear contradictory as there is a decrease in the volume of vigorous PA; however, we believe this reduction was compensated for by higher levels of other PA and the increase in age. The children in this context also started engaging in non-sports extracurricular activities, consequently increasing time dedicated to schoolwork.

Overall, over the three years considered, weekly vigorous PA averaged 3.40 ± 1.55 h/week, not accounting for the two hours per week of mandatory physical education within the school context. In total, 5.40 h/week of PA were achieved for the children. This suggests that the time spent on PA falls within the parameters considered as healthy PA, as recommended by the World Health Organization (2018) (34), which suggested at least 1 h of moderate to vigorous PA per day for children and adolescents.

The PA conducted in the M.A.M.I. Deporte programme also aligned with recommendations for school-age children and adolescents by Janssen and LeBlanc (35), stating that children and youth aged 5 to 17 should accumulate an average of at least 60 min per day and up to several hours of moderate-intensity PA. Some health benefits can also be achieved with an average of 30 min per day, especially when engaging in vigorous PA, which is crucial for preventing and treating childhood and adolescent obesity based on evidence-based guidelines (36).

The M.A.M.I Deporte programme also included longer intensive activities, as suggested by some authors (37), who recommend incorporating them as soon as possible, at least three days a week, with the majority being aerobic activities.

The type and intensity of activities in the M.A.M.I. Deporte programme are not consistently practiced in all settings. In the United States, less than half of children from various social, ethnic, and family backgrounds engage in≥60 min/day of moderate to vigorous PA (38). In Spain, a high percentage of students regularly participate in extracurricular sports (67.6%), with boys leaning toward competitive team sports (soccer or basketball) and girls favoring gymnastic sports (39).

However, there is a high rate of dropout among children from one academic year to the next, often due to difficulties parents encounter in participating in sports activities (40). Children living in environments that promote extracurricular sports activities, nutritional programs, and encourage parental involvement improve their overall PA levels and healthy behaviors compared to children in controlled environments (41). Therefore, action is needed not only on children but also on the environment.

The mean age of our participants was 6.3 ± 2.3 years. There were very few participants over 10 years old, so our influence on the critical age at which adherence to recommendations for vigorous PA drastically decreases (42) was limited. This age is also when children start compulsory secondary education. However, other subgroups at risk of inactivity include older girls and adolescents, among others (43). In our activity, the percentage of female participants was 48.31%, indicating that the activities were not strongly biased by gender.

In some programs for initiating children into sports, besides planning sports activities adapted for children and parents, other health-related behaviors have been developed, such as promoting proper nutrition (44). Previous qualitative research on parents' perceptions of healthy eating and PA among preschool-age children emphasizes the need to develop and implement effective programs for preschool-age children and their caregivers (45). The M.A.M.I. Deporte programme has been based on the concept of the Healthy Habits Program (46), which can help improve childhood nutrition and health. Understanding how children think about food choices can enhance our understanding of nutrition knowledge and dietary behaviors in children, helping to comprehend conflicting pressures influencing children's healthy lifestyles and improve communication on these topics among parents, educators, coaches, and children (47). In this regard, the M.A.M.I. Deporte programme also facilitated healthy snacks after sports activities, aiming to implement a multicomponent intervention that positively influenced PA participation from both the program and family perspectives.

The high prevalence of physical inactivity and obesity in children and adolescents has become a global problem (48, 49, 50). So far a large majority of studies have observed that there is a weak positive relationship between parent and child PA, irrespective of the age of the child, the sex of the parent-child dyad and the type of PA (46). In this context, PA of preschool children and its specific implementation in kindergartens and families is the most complex and responsible pedagogical process (47). Moreover, in our socio-economic context, it is a priority to organise joint educational and sports processes, involving trained personnel in monitoring and assessing children's health.

Recent studies indicate that gender, age, ethnicity and self-concept are the most common influencing factors on the intrapersonal level (51). At the interpersonal and organisational levels, support from friends, parents and teachers are positive predictors of children's participation in PA (49). Accessibility to safe facilities and neighbourhoods is also a crucial factor that can influence children's and adolescents' participation in PA at the community level (52).

A limitation of the study is the absence of a control group from the same environment and social range as the participants. However, studies on the Spanish population in general indicate that the majority of children do not meet the minimum recommended levels of PA (40). For future directions, it would be crucial for our methodology to be supported by policies promoting PA to enhance implementation and adherence and encourage children's participation in both organized and recreational physical activities. Another limitation is that we may have incurred a possible selection bias of the participating families that may not be representative of the general population, affecting external validity on the understanding that the findings may not be extrapolated to the general population.

Although parental intervention has been well accepted as a possible mechanism for children's participation in PA (53), there is a weak correlation between parents' and children's level of PA and there may be a need to develop a deeper understanding of the associations and mechanisms that influence children's PA practice. Other dimensions that could be examined would be the types of home and school activity, such as sitting, standing, running during children's play and cycling. To broaden the understanding of PA in the family context, it could also be fruitful to consider siblings.

Finally, from an operational point of view, the MAMI Deporte programme used the multi-sports facilities of a top level higher education institution. This availability of means may not be found in other sport contexts, limited to a few sport disciplines, and may constitute a barrier to enhance multiple motor skills of children and parents in less favoured sport contexts.

In conclusion, the active methodology for improving sports initiation (M.A.M.I. Deporte) shared between children and parents successfully initiates children into sports activities, maintains their activity, and promotes long-term adherence, as the rate of incorporation into organized sports activities was higher than the rate of dropouts. Developing methodologies that encourage joint participation of parents and children would undoubtedly improve the PA habits of children and, in the long run, the population.

### Practical applications

From our point of view, society demands that the role and responsibilities of the family in the education of children be extended towards healthy exercise in line with the demands of the times. From a practical point of view, developing PA programmes involving parents and children can promote sports activities in the family environment and share healthy lifestyle habits. It is also important to develop a better understanding of parents' influence on children's PA in order to identify effective strategies to increase children's PA and contribute to enhancing positive effects on health and well-being.

## Data Availability

The raw data supporting the conclusions of this article will be made available by the authors, without undue reservation.
