# Supplementary material for: Efficacy of physical activity shared between parents and children to improve sports initiation in the M.A.M.I.deporte® program
Source: Front Sports Act Living. 2024 Mar 26;6:1372664. doi: 10.3389/fspor.2024.1372664 (PMC11002184; doi:10.3389/fspor.2024.1372664)
Supplement: Supplementary file 1 [file Datasheet1.pdf]

SUPPLEMENTARY MATERIAL

Repeated Measures Anova

**Anova**					**Alpha**	**0,05**
Sources	***SS***	***df***	***MS***	***F***	***P value***	***P Eta-sq***
Subjects	212.04	33.00	6.43	3.58	0.00	0.64
Groups	4.29	2.00	2.15	1.20	0.31	0.04
Error	118.37	66.00	1.79			
Total	334.71	101.00				

**Greenhouse and Geisser**					**Alpha**	**0.05**
Sources	***SS***	***Df***	***MS***	***F***	***P value***	***P Eta-sq***
**Groups**	4.29	1.97	2.18	1.20	0.31	0.04
**Error**	118.37	65.07	1.82			

Huyhn and Feldt					***Alpha***	***0.05***
Sources	***SS***	***Df***	***MS***	***F***	***P value***	***P Eta-sq***
Groups	4.29	2.00	2.15	1.20	0.31	0.04
Error	118.37	66.00	1.79			

Note: **SS**: Sum-of-squares; **Df**: Degrees of freedom; **MS**: Mean squares; **F**: F ratio; **P Eta-sq**: Partial Eta Squared.

Pairwise paired t tests

group 1	group 2	p-value	Mean
**Year 1**	**Year 2**	0.52	0.21
**Year 1**	**Year 3**	0.16	0.50
**Year 2**	**Year 3**	0.36	0.29
