# Supplementary material for: Efficacy of physical activity shared between parents and children to improve sports initiation in the M.A.M.I.deporte® program
Source: Front Sports Act Living. 2024 Mar 26;6:1372664. doi: 10.3389/fspor.2024.1372664 (PMC11002184; doi:10.3389/fspor.2024.1372664)
Supplement: Supplementary file 2 [file Datasheet2.docx]

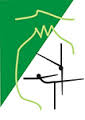
 [
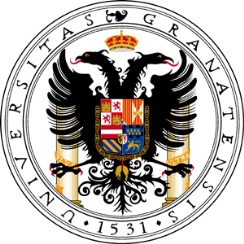
](http://www.google.es/url?sa=i&rct=j&q=&esrc=s&frm=1&source=images&cd=&cad=rja&docid=3Po2lVUjTA3yQM&tbnid=nFwCYdwXZR4mNM:&ved=0CAUQjRw&url=http://www.bellasartesgranada.org/index.php/Detalle-de-noticia/147/0/?&tx_ttnews%5btt_news%5d=822&tx_ttnews%5bbackPid%5d=24&cHash=d1f1163ad9&ei=Ap2sUrSEOIPX0QWm24DQBw&bvm=bv.57967247,d.d2k&psig=AFQjCNFI9YsUiXKN3ly8LkI67cMUKYd8bQ&ust=1387130456431386)

**Survey for users of the project**

**"Active Methodology for the Improvement of Sport Initiation:**

**M.A.M.I. Deporte".**

1. Indicate with an "X" what you think of the activities you have performed:

|  | More fun | More difficult | Less fun | Less difficult |
| --- | --- | --- | --- | --- |
| Presentation games |  |  |  |  |
| Games with music |  |  |  |  |
| Warm-up games |  |  |  |  |
| Handball |  |  |  |  |
| Basketball |  |  |  |  |
| Hockey Indoor |  |  |  |  |
| Football |  |  |  |  |
| Racquetball |  |  |  |  |
| Athletics |  |  |  |  |
| Judo |  |  |  |  |
| Volleyball |  |  |  |  |

1. Indicate with an "X" other activities you would like to do:

|  | For parents | For children | The whole family |
| --- | --- | --- | --- |
| Swimming |  |  |  |
| Rhythmic Gymnastics |  |  |  |
| Artistic Gymnastics |  |  |  |
| Choreography |  |  |  |
| Rugby |  |  |  |
| Traditional folk games |  |  |  |
| Other (please tick the appropriate box): |  |  |  |
|  |  |  |  |
|  |  |  |  |

3.- How often would you be willing to do the M.A.M.I. Sport activity next year?

Times per week

| 1 | 2 | 3 |
| --- | --- | --- |
|  |  |  |

4.- Prior to this activity, was your child involved in any other sporting activity?

|  |  | Boy 1 (age..........) | Girl 1 (age..........) |
| --- | --- | --- | --- |
| Activity:  ……………... | Hours per week |  |  |
|  | Length of time you have been doing this activity (months, years) |  |  |
| Activity:  ……………... | Hours per week |  |  |
|  | Length of time you have been doing this activity (months, years) |  |  |
| Activity:  …………… | Hours per week |  |  |
|  | Length of time you have been doing this activity (months, years) |  |  |

|  |  | Boy 1 (age..........) | Girl 1 (age..........) |
| --- | --- | --- | --- |
| Activity:  ……………... | Hours per week |  |  |
|  | Length of time you have been doing this activity (months, years) |  |  |
| Activity:  ……………... | Hours per week |  |  |
|  | Length of time you have been doing this activity (months, years) |  |  |
| Activity:  …………… | Hours per week |  |  |
|  | Length of time you have been doing this activity (months, years) |  |  |

|  |  | Boy 1 (age..........) | Girl 1 (age..........) |
| --- | --- | --- | --- |
| Activity:  ……………... | Hours per week |  |  |
|  | Length of time you have been doing this activity (months, years) |  |  |
| Activity:  ……………... | Hours per week |  |  |
|  | Length of time you have been doing this activity (months, years) |  |  |
| Activity:  …………… | Hours per week |  |  |
|  | Length of time you have been doing this activity (months, years) |  |  |

5.- Prior to this activity, was your child involved in any other NON-sport activity?

|  |  | Boy 1 (age..........) | Girl 1 (age..........) |
| --- | --- | --- | --- |
| Activity:  ……………... | Hours per week |  |  |
|  | Length of time you have been doing this activity (months, years) |  |  |
| Activity:  ……………... | Hours per week |  |  |
|  | Length of time you have been doing this activity (months, years) |  |  |
| Activity:  …………… | Hours per week |  |  |
|  | Length of time you have been doing this activity (months, years) |  |  |

|  |  | Boy 1 (age..........) | Girl 1 (age..........) |
| --- | --- | --- | --- |
| Activity:  ……………... | Hours per week |  |  |
|  | Length of time you have been doing this activity (months, years) |  |  |
| Activity:  ……………... | Hours per week |  |  |
|  | Length of time you have been doing this activity (months, years) |  |  |
| Activity:  …………… | Hours per week |  |  |
|  | Length of time you have been doing this activity (months, years) |  |  |

|  |  | Boy 1 (age..........) | Girl 1 (age..........) |
| --- | --- | --- | --- |
| Activity:  ……………... | Hours per week |  |  |
|  | Length of time you have been doing this activity (months, years) |  |  |
| Activity:  ……………... | Hours per week |  |  |
|  | Length of time you have been doing this activity (months, years) |  |  |
| Activity:  …………… | Hours per week |  |  |
|  | Length of time you have been doing this activity (months, years) |  |  |

6- During your attendance to M.A.M.I. Sport have you taken part in other sport activities?

|  |  | Boy 1 (age..........) | Girl 1 (age..........) |
| --- | --- | --- | --- |
| Activity:  ……………... | Hours per week |  |  |
|  | Length of time you have been doing this activity (months, years) |  |  |
| Activity:  ……………... | Hours per week |  |  |
|  | Length of time you have been doing this activity (months, years) |  |  |
| Activity:  …………… | Hours per week |  |  |
|  | Length of time you have been doing this activity (months, years) |  |  |

|  |  | Boy 1 (age..........) | Girl 1 (age..........) |
| --- | --- | --- | --- |
| Activity:  ……………... | Hours per week |  |  |
|  | Length of time you have been doing this activity (months, years) |  |  |
| Activity:  ……………... | Hours per week |  |  |
|  | Length of time you have been doing this activity (months, years) |  |  |
| Activity:  …………… | Hours per week |  |  |
|  | Length of time you have been doing this activity (months, years) |  |  |

|  |  | Boy 1 (age..........) | Girl 1 (age..........) |
| --- | --- | --- | --- |
| Activity:  ……………... | Hours per week |  |  |
|  | Length of time you have been doing this activity (months, years) |  |  |
| Activity:  ……………... | Hours per week |  |  |
|  | Length of time you have been doing this activity (months, years) |  |  |
| Activity:  …………… | Hours per week |  |  |
|  | Length of time you have been doing this activity (months, years) |  |  |

7.- Assessment of activity:

| Training of monitors | 1 | 2 | 3 | 4 | 5 | 6 | 7 | 8 | 9 | 10 |
| --- | --- | --- | --- | --- | --- | --- | --- | --- | --- | --- |
| Performance of tasks | 1 | 2 | 3 | 4 | 5 | 6 | 7 | 8 | 9 | 10 |
| Methodology of the classes | 1 | 2 | 3 | 4 | 5 | 6 | 7 | 8 | 9 | 10 |
| Conditions of the facilities | 1 | 2 | 3 | 4 | 5 | 6 | 7 | 8 | 9 | 10 |
| Outcomes obtained by your child | 1 | 2 | 3 | 4 | 5 | 6 | 7 | 8 | 9 | 10 |
| Adequacy of the activity to my child's needs | 1 | 2 | 3 | 4 | 5 | 6 | 7 | 8 | 9 | 10 |
| Adequacy of the activity to my child's needs | 1 | 2 | 3 | 4 | 5 | 6 | 7 | 8 | 9 | 10 |
| In general, the level of satisfaction is… | 1 | 2 | 3 | 4 | 5 | 6 | 7 | 8 | 9 | 10 |

8.- What suggestions would you make to make the activity more enjoyable?

**THANK YOU VERY MUCH!!!**
